# Evaluation of Outcomes in Immature Teeth After Revitalization or Apexification Procedures: A Systematic Review and Meta-Analysis

**DOI:** 10.7759/cureus.60357

**Published:** 2024-05-15

**Authors:** Marilena Stefanidou, Ana Kostenkova, Jolanta Siudikienė, Greta Lodienė

**Affiliations:** 1 Faculty of Odontology, Lithuanian University of Health Sciences, Kaunas, LTU; 2 Department of Dental and Oral Pathology, Lithuanian University of Health Sciences, Kaunas, LTU

**Keywords:** dentin thickness, root length, pulp necrosis, root canal therapy, immature teeth, regenerative endodontics therapy

## Abstract

There are two main treatment options for immature teeth with necrotic pulp and apical periodontitis. Apexification creates a mineralized tissue barrier, while revitalization aims to regenerate vital tissue in the canal space. There is no conclusive evidence to determine the most effective procedure regarding root length and dentin wall thickness. The objective of this systematic review was to compare the outcomes of revitalization and apexification procedures in immature non-vital teeth in terms of root length and dentin wall thickness.

A literature search was conducted using the PubMed, ScienceDirect, Google Scholar, and Embase databases. Articles relevant to the study topic were gathered according to the selection criteria, following the Preferred Reporting Items for Systematic Reviews and Meta-Analyses guidelines. The included studies had to be published in English, conducted over a six-year period, and compared the outcomes of revitalization and apexification procedures in immature non-vital teeth. Data were collected using appropriate keywords from the eligible studies.

Six articles were included for qualitative and quantitative analysis. The eligible studies showed a low risk of bias. In all revitalization cases, the root length increased significantly (mean difference (MD) (%) = 5.91; 95% confidence interval (CI) = 2.39-9.43; p = 0.0010; MD (mm) = 2.43; 95% CI = 2.05-2.80; p < 0.00001). The dentin wall thickness was statistically significant in most cases (MD (%) = 10.94; 95% CI = 7.01-14.88; p < 0.00001), MD (mm) = 0.16; 95% CI = 0.07-0.25; p = 0.0007).

The systematic review and meta-analysis showed both procedures to be credible treatment options for necrotic immature teeth. Apexification had a positive impact, to some extent, on the development of root length. Revitalization yielded a significantly greater increase in root length and root dentin wall thickness and appeared to be superior in promoting root development.

## Introduction and background

The treatment of immature teeth with necrotic pulp and apical periodontitis poses multiple challenges to achieving a positive outcome [1]. These challenges include a greater risk of complications that make the tooth root prone to fractures after the treatment [2], as well as a more complicated root canal disinfection and obturation [1]. Current treatment modalities for non-vital immature permanent teeth include apexification and revitalization procedures [3].

Teeth with necrotic pulp and/or apical periodontitis were traditionally treated using the apexification method to induce a mineralized tissue barrier in immature teeth. With the introduction of hydraulic calcium silicate cements (HCSCs), they have become the material of choice in apexification procedures [4]. An apical plug can be created using mineral trioxide aggregate (MTA) [5] or other HCSCs [4] promptly before root canal obturation, reducing the number of clinical visits required. However, apexification has no potential to restore the vitality of damaged tissue in the root canal space. As a result, it fails to promote the development and maturation of immature non-vital permanent teeth [6].

Revitalization, in contrast, is a concept where the vital tissue in the canal space damaged by infection, trauma, or development anomalies is regenerated [6]. Regenerative endodontic procedures (REPs) were defined by Murray et al. (2007) as “biologically based procedures designed to replace damaged structures, including dentine and root structures as well as cells of the pulp-dentin complex” [7]. The primary goal of REPs is to create a conductive environment in the root canal that promotes differentiation of mesenchymal stem cells, regeneration of pulpal tissue, and root development continuation. REP is an alternative to apexification with similar objectives but based on biological tissue properties. Successful REP results in root elongation, dentin wall thickening, and apex closure, which is essential for the future prognosis of immature teeth [7]. The European Society of Endodontology (ESE) provided two appointments’ procedure REP guidelines with short-term calcium hydroxide intracanal medication following the induction of a blood clot (BC) into the root canal space and placing HCSC above [8]. The American Association of Endodontists (AAE) recommends using calcium hydroxide or a low concentration of triple antibiotic paste (TAP) as an intracanal medication in the REP [9].

Some studies compared the two techniques of non-vital immature permanent teeth treatment. Several systematic reviews concluded that REP and apexification had equal success rates in treating necrotic immature teeth [2,10,11]. Nevertheless, there is no clear evidence about the most effective procedure in terms of root length and dentin wall thickness. Consequently, the objective of this systematic literature review and meta-analysis was to evaluate the long-term outcomes in terms of root length and dentin wall thickness of apexification and revitalization procedures to compare the effect of appropriate treatment methods.

## Review

Methodology

Protocol and Registration

This systematic review followed the Preferred Reporting Items for Systematic Reviews and Meta-analyses (PRISMA) guidelines [11,12]. The study protocol was registered at the International Prospective Registry of Systematic Reviews (PROSPERO) database (registration number: CRD42023482116).

Eligibility Criteria and Selection Process

The focus question of this review, “Which procedure: apexification or revitalization outcomes are more appropriate for the treatment of non-vital immature permanent teeth, in terms of root length and root dentin wall thickness?,” was prepared following the Participant, Intervention, Comparison, Outcome, Time (PICOT) principle [13]. In the included studies, the patients free of systematic medical illnesses diagnosed with necrotic pulp and/or apical periodontitis in immature permanent teeth (P) received endodontic treatment (I). The treatment included either revitalization or apexification procedures (C). Only studies comparing the outcomes in the changes of root length and dentin thickness (O) following both interventions were eligible for inclusion. In all included studies, a minimum of six months for all outcome measures was observed (T). Included studies were randomized controlled trials, retrospective, and prospective studies published between 2017 and 2023 in English. The review excluded studies on mature or primary teeth, animals, in vitro studies, case reports and case series, studies without follow-up periods, articles that could not be accessed or downloaded, articles with content in languages other than English, and articles lacking comprehensive details.

This systematic review focused on the selection and identification of information from PubMed/PMC, ScienceDirect, Google Scholar, and Embase electronic databases from November 10, 2023, until November 31, 2023. Electronic scientific databases were searched based on PRISMA guidelines [10,11]. A detailed analysis of scientific electronic data was conducted independently by two investigators (MS, AK) using a variety of keyword combinations to acquire the optimal research data. The full keywords and their combinations used for the search on these selected databases are presented in Table 1.

**Table 1:** Strategies for database search.

Database	Search strategy
PubMed/PMC	#1 “Revitalization” [All Fields] OR “Regeneration” [All Fields] OR “Revascularization” [All Fields] OR “Pulp revitalization” [All Fields] OR “Pulp Regeneration” [All Fields] OR “Pulp Revascularization” [All Fields]; #2 “Apexification” [All Fields] OR “Apexification procedure” [All Fields]; #3 “Pulp necrosis” [All Fields] OR “Non-vital tooth” [All Fields] OR “Non vital” [All Fields]; #4 “Apical periodontitis” [All Fields]; #5 “Permanent” [All Fields] OR “Permanent tooth” [All Fields] OR “Immature tooth” [All Fields] OR “Immature” [All Fields]; Search combination performed was #1 AND #2 AND #3 OR #1 AND #2 AND #4 OR #1 AND #2 AND #5
ScienceDirect	
Google Scholar	
Embase	

**Figure 1:**
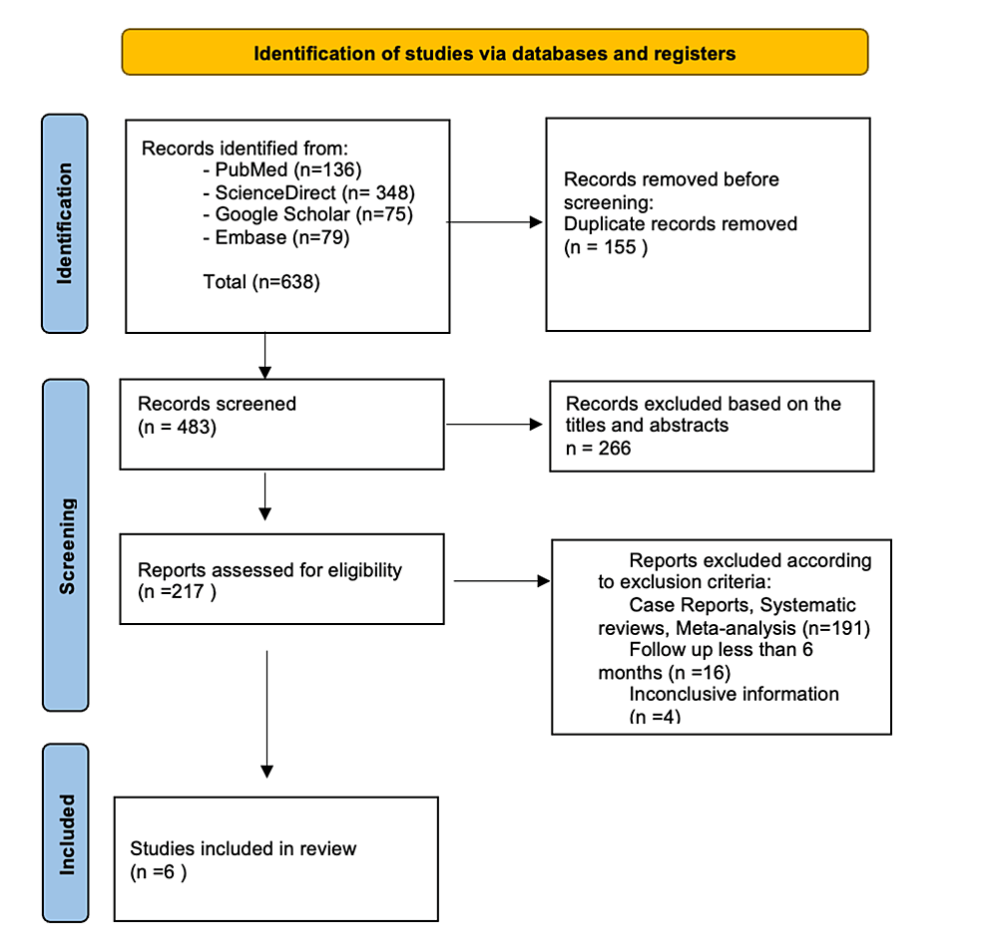
PRISMA flowchart outlining the search strategy. PRISMA = Preferred Reporting Items for Systematic Reviews and Meta-Analyses

Two investigators (MS, AK) independently analyzed the titles and abstracts of the studies that met the eligibility criteria. Duplicate records were eliminated, and the remaining articles were screened according to the inclusion and exclusion criteria. The full texts were then evaluated according to the eligibility criteria by the same group of authors. Any disagreements were resolved by discussion with the third author, a senior researcher in endodontology (GL). The publications were assessed further for their relevance and eligibility. This review followed the PRISMA statement guidelines, as indicated in Figure 1.

Data Extraction and Statistical Analysis

Two independent authors (MS, AK) extracted data and collected the following principal characteristics from each included study: (a) the authors and year of publication, (b) the study type, (c) the age range of the participants, (d) the tooth type and number of teeth included, (e) the etiology of the examined teeth, (f) the diagnosis of the teeth receiving either treatment, (g) the materials and the protocols used in each procedure, (h) the measured outcomes, and (i) the follow-up periods. A meta-analysis was conducted, in cases where adequate data was provided, using the Review Manager (RevMan) (Cochrane Collaboration, Oxford, UK). The heterogeneity of each study was determined using the I^2^ test. According to the guidelines presented in the Cochrane Handbook for Systematic Reviews of Interventions, the values of the I^2^ test were interpreted as follows: a value less than 40% was considered as heterogeneity that might not be important; 30% to 60% may represent moderate heterogeneity; 50% to 90% may represent substantial heterogeneity; and 75% to 100% presents considerable heterogeneity [14]. P-values <0.05 were considered statistically significant. The inverse variance (IV) statistical method was used to compare the different continuous outcome variables. A fixed-effects model was employed in the presence of low heterogeneity [15]. Fixed- and random-effect models were presented as forest plots with 95% confidence intervals (CIs). The effect size between the two outcome groups was demonstrated as a mean difference (MD).

Risk of Bias Assessment

The Joanna Briggs Institute (JBI) critical appraisal tools [16] were used to assess the risk of bias among the six articles (three randomized controlled studies and three retrospective cohort studies) that were included in this systematic review. The JBI appraisal tool allocated a simple “Yes,” “No,” “Unclear,” or “Not Applicable” to each of the checklist’s questions. This evaluation aimed to assess the quality of the publications included in this review. All incorporated articles were subjected to an extensive evaluation by critical appraisers.

Results

Study Characteristics and Quality Assessment

The quality of the included studies was assessed using the JBI critical appraisal tool [16]. Six studies [17-22] were included, three were evaluated according to the checklist for randomized controlled trials, and three according to the checklist of cohort studies, as seen in Table 2 and Table 3, respectively. The overall risk of bias among the incorporated studies was assessed. As all studies met most of the criteria of the checklist questions, and the assessment of bias in study design, conduct, and analysis were described, it was concluded that the selected studies for this review were of good quality and the risk of bias was low [15].

**Table 2:** JBI critical appraisal checklist for randomized controlled trials. JBI = Joanna Briggs Institute; RCT = randomized controlled trial; + = yes; - = no; / = unclear

Checklist	Author (year)		
	Lin et al. (2017) [19]	Xuan et al. (2018) [21]	Awies et al. (2017) [17]
Was true randomization used for the assignment of participants to treatment groups?	+	+	+
Was allocation to treatment groups concealed?	+	+	+
Were treatment groups similar at the baseline?	+	+	+
Were participants blind to treatment assignment?	+	+	+
Were those delivering treatment blind to treatment assignment?	/	+	/
Were outcome assessors blind to treatment assignment?	/	+	/
Were treatment groups treated identically other than the intervention of interest?	+	+	+
Was follow-up complete, and if not, were differences between groups in terms of their follow-up adequately described and analyzed?	+	+	+
Were participants analyzed in the groups to which they were randomized?	+	+	+
Were outcomes measured in the same way for treatment groups?	+	+	+
Were outcomes measured reliably?	+	+	+
Was appropriate statistical analysis used?	+	-	+
Was the trial design appropriate, and any deviations from the standard RCT design (individual randomization, parallel groups) accounted for in the conduct and analysis of the trial?	+	+	+

**Table 3:** JBI critical appraisal checklist for cohort studies. JBI = Joanna Briggs Institute; + = Yes; - = No; / = unclear; N/A = not applicable

Checklist	Author (year)		
	Caleza- Jiménez et al. (2022) [20]	Cardoso Pereira et al. (2021) [18]	Silujjai et al. (2017) [22]
Were the two groups similar and recruited from the same population?	+	+	+
Were the exposures measured similarly to assign people to both exposed and unexposed groups?	N/A	N/A	N/A
Was the exposure measured in a valid and reliable way?	+	+	+
Were confounding factors identified?	+	+	/
Were strategies to deal with confounding factors stated?	+	+	/
Were the groups/participants free of the outcome at the start of the study (or at the moment of exposure)?	+	+	+
Were the outcomes measured in a valid and reliable way?	+	+	+
Was the follow-up time reported and sufficiently long enough for outcomes to occur?	+	+	+
Was follow-up complete, and if not, were the reasons for loss to follow-up described and explored?	+	+	+
Were strategies to address incomplete follow-up utilized?	+	+	+
Was appropriate statistical analysis used?	+	+	+

The characteristics of each study are summarized in Table 4. All studies were assessed for outcomes of interest, where the main cause for pulp necrosis or apical periodontitis was either dental trauma, caries, or dental anomalies, i.e., dens evaginatus. The diagnosis and the assessment of each outcome were based on the clinical and radiographic evaluation. A total of 260 non-vital immature permanent teeth were evaluated with follow-up periods ranging from 6 to 24 months [17-22].

**Table 4:** Principal characteristics of the included studies. REP = regenerative endodontic procedure; TAP = triple antibiotic paste; BC = blood clot; MTA = mineral trioxide aggregate; CH = calcium hydroxide; GP = gutta-percha; CHX = chlorohexidine; hDPSC = human deciduous pulp stem cells

Authors (publication year)	Country	Study type	Age range (years)	Tooth type and number (n)	Etiology	Diagnosis	Materials	Outcomes	Follow-up Period
Lin et al. (2017) [19]	China	Prospective randomized controlled study	6–18	Permanent premolar (REP: 48, apexification: 21), permanent central incisor (REP: 21, apexification: 13). Total (n): 103 REP: 69, apexification: 34	Trauma, dens evaginatus	Pulp necrosis in immature teeth	REP: TAP + BC + absorbable collage barrier + MTA apexification: CH + GP	At 12 months: root length: REP: increased apexification: increasing. Root dentin wall thickness: REP: increased apexification: increased	3, 6, 9, 12 months
Caleza-Jiménez et al. (2022) [20]	Spain	Retrospective study	7–10 (mean age: 8 ± 1.04)	Incisors (12), molars (6), total (n): 18, REP: 9, apexification: 9	Trauma (66.6%), caries (33.3%)	Necrotic immature permanent teeth	REP: TAP + BC+ MTA, apexification: CH + MTA + GP	At 6 months: root length: REP: increased apexification: increasing. Root dentin wall thickness: REP: increased apexification: decreased	6–66 months (mean: 22 months)
Pereira et al. (2021) [18]	Brazil	Retrospective study	6–18	Upper central incisor (43), upper lateral incisor (1), total (n): 44, REP: 22, apexification: 22	Trauma	Pulp necrosis/necrotic immature permanent teeth	REP: TAP or CH/CHX + BC + collagen fibres + MTA, apexification: CH + GP	At 12–30 months: root length: REP: increased, apexification: increased. Root dentin wall thickness: REP: increased apexification: increased	REP: 12–30 months, apexification: 12–24 months
Xuan et al. (2018) [21]	China	Prospective randomized controlled study	7–12	Incisors, total (n): 30, REP: 20, apexification: 10	Trauma	Pulp necrosis and apical periodontitis in immature permanent teeth	REP: hDPSC implantation apexification: CH	At 12 months: root length: REP: increased apexification: increased. Root dentin wall thickness: REP: increased apexification: no change	3, 6, 9, 12 months
Silujjai et al. (2017) [22]	Thailand	Retrospective study	8–46 (median age: 13)	Central incisors, 1st and 2nd premolars, 1st and 2nd molars, total (n): 43 REP: 17 apexification: 26	Trauma (46.51%), dens evaginatus (41.86%), caries (11.68%)	Non-vital immature teeth	REP: CH or TAP + BC + MTA, apexification: CH + MTA + GP	At 12-96 months: Root length: REP: increased apexification: increased. Root dentin wall thickness: REP: increased apexification: decreased	6, 12 months, 2, 3, 4, 5 years
Awies et al. (2017) [17]	Egypt	Prospective randomized controlled study	8–17	Upper and lower anterior teeth, total (n): 22 REP: 11 apexification: 11	Trauma	Necrotic immature permanent teeth	REP: TAP + BC + MTA, apexification: TAP + MTA	At 9 months: root length: REP: increased apexification: increased. Root dentin wall thickness: REP: increased apexification: increased	1, 3, 6, 9 months

The review of endodontic treatment used in these studies showed that, initially, all immature teeth were treated by a similar endodontic protocol. All patients were anesthetized with either 2% lidocaine [18,19] or 3% mepivacaine [16]; the anesthetic solution was not specified in some cases [20-22]. After the administration of the local anesthetic solution, a rubber dam was placed, an access cavity was prepared, and the working length was determined [17-20,22]. Each canal was irrigated with sodium hypochlorite solution with a concentration ranging from 1.5-2.5% [19,20,22], 5.25% [17], or 6% [18] depending on the case. In most studies, a 17% ethylenediaminetetraacetic acid (EDTA) solution was also used [18,19,22].

The entirety of the revitalization cases was treated in two visits. During the first visit, and after the initial tooth preparation, a triple antibiotic paste (TAP) mixture [17-20,22] or calcium hydroxide (CH) paste [18,22] was placed into the canals, followed by a temporary restoration. The second visit typically occurred after three weeks [17-19], where the same preparation protocol was performed. The TAP mixture was removed from the canal and a final irrigation with 17% EDTA was performed [18,19]. Using paper points, some canals were dried [17,19], and in most cases, a BC was induced into the root canal reaching 3-4 mm below the cementoenamel junction [17,19], followed by white or gray MTA [17-19,21,22] with an approximate thickness of 2-3 mm [17,18,20,22].

According to the analysis of all the reviewed studies, the apexification treatment reported using a two-visit approach [17-22]. During the first visit, CH paste was placed as an intracanal medicament for approximately one week [19]. In certain cases, MTA was also placed as a temporary filling of the accessed cavity [18,20,22]. Only in the study conducted by Awies et al., TAP paste was placed instead of CH [17]. After three weeks [17-19], when confirmation of apical barrier formation was done, the CH paste, MTA, or TAP mixture was removed at the second visit, the canals were irrigated with sodium hypochlorite solution [17,22] or 17% EDTA and saline solution [18,19], and final obturation with warm/injectable gutta-percha was completed [17-20]. In most cases, temporary restoration with glass ionomer cement [17,19,21] and composite resin was the final step [17-20,22].

The study by Xuan et al. (2018) differed in multiple aspects. At the first visit, conventional endodontic disinfection was performed for all 30 patients [21]. During the second visit, which occurred one month later, 20 patients, randomly assigned to undergo REP treatment, had pulp extirpated from their maxillary deciduous canines. Subsequently, human deciduous pulp stem cell (hDPSC) aggregates were implanted into the pulp canal space of the traumatized incisor. Following the same timeline, the remaining patients received the apexification treatment. The exact protocol followed for the treatment with the apexification procedure was not stated clearly. However, the author mentioned that the 10 control patients received traditional apexification cone-beam computed tomography (CBCT) treatment.

Radiographic evaluation was performed pre and postoperatively in all cases. Periapical radiographs were obtained in most studies [17-20,22] while CBCT imaging was used only in certain studies [19,21]. The periapical radiographs taken in the gathered studies used either the Rinn XCP alignment system (Densply Sirona, Charlotte, North Carolina, USA) [17,20,22] or a receptor-holding instrument [18]. The radiographs taken pre-treatment were compared to the ones taken at the follow-up visits. All studies reported their findings in terms of root length and dentin wall thickness at the baseline and following a designated follow-up period.

Synthesis of Results

The summary results of each study are presented in Table 5. All teeth that underwent REP or apexification procedures showed an increase in root length. In addition, in all six studies, the root dentin wall thickness of teeth treated with REP increased [17-22]. Only three studies showed an increase in root dentin wall thickness after the apexification procedure [17-19], Caleza-Jiménez et al. [20] showed a decrease in root dentin wall thickness, and Silujjai et al. [22] showed a decrease in root dentin wall thickness, while Xuan et al. showed no change in their study [21].

**Table 5:** Root development parameters: root length and root dentin wall thickness (mean values ± standard deviation (SD) or percentage (%) after the follow-up period). *: Xuan et al. [21] study reported only qualitative data on root thickness parameters. REP = regenerative endodontic procedures

Authors (year, country of the study)	Root length		Root dentin wall thickness	
	REP	Apexification	REP	Apexification
Lin et al. (2017, China) [19]	1.64 ± 1.43 mm	0.60 ± 1.06 mm	0.24 ± 0.25 mm	0.08 ± 0.21 mm
Caleza-Jiménez (2022, Spain) [20]	12.76 ± 10.15%	0.29 ± 0.59%	34.57 ± 16.62%	-3.36 ± 4.13%
Pereira et al. (2021, Brazil) [18]	12.55 ±1 1.89%	6.66 ± 5.28%	6.70 ± 11.08%	0.99 ± 2.25%
Xuan et al. (2018, China) [21]	5.24 ± 0.92 mm	0.88 ± 0.67 mm	Increase*	No increase*
Silujjai et al. (2017, Thailand) [22]	9.51 ± 18.14%	8.55 ± 8.97%	13.75 ± 19.91%	-3.30 ± 14.14%
Awies et al. (2017, Egypt) [17]	5.00 ± 9.72 mm	6.17 ± 11.36 mm	6.07 ± 25.54%	4.65 ± 13.66%

Meta-analysis

All six studies were included in the meta-analysis. The fixed-effects model was used in the cases where the heterogeneity was not deemed significant, and/or it was reasonable to consider that all studies were similar enough so that there was a common effect [15]. Forest plots were used to display graphically the summarized findings of the meta-analysis, as they provided the necessary information to facilitate the interpretation of the outcomes. MD used in the meta-analysis of continuous data of all the integrated studies measured the same outcome on the same measurement unit [23]. MD was used over standardized mean difference as it was preferable when studies in a meta-analysis measured the outcomes using the same scales and/or instruments. A meta-analysis was performed to compare the treatment outcomes between revitalization and apexification procedures, where four forest plots were created.

Root Length

**Figure 2:**
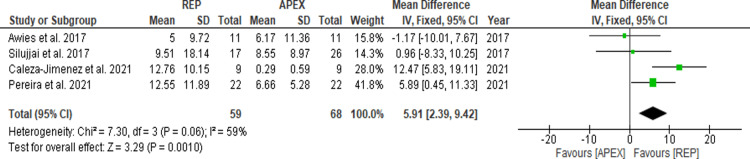
Root length after APEX and REP (%). Awies et al. (2017) [17]; Silujjai et al. (2017) [22]; Caleza-Jimenez et al. (2021) [20]; Pereira et al. (2021) [18]. REP = regenerative endodontic procedure; APEX = apexification procedure

**Figure 3:**
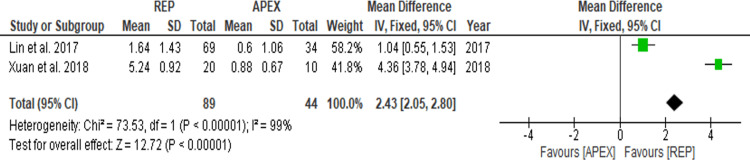
Root length after APEX and REP (mm). Lin et al. (2017) [19], Xuan et al. (2018) [21]. REP = regenerative endodontic procedure; APEX = apexification procedure

Four of the included studies reported the root length measurements as a percentage (%) [17,18,20,22], while the remaining two [19,21] used millimeters (mm) as the measurement unit. Therefore, two different data analyses and forest plots were created. Figure 2 and Figure 3 illustrate the forest plots of the root length outcomes. The MD in the root length (%) was 5.91 (95% CI = 2.39-9.43; p = 0.0010), while the MD in root length (mm) was 2.43 (95% CI = 2.05-2.80; p < 0.00001), both of which were deemed statistically significant.

Root Dentin Wall Thickness

**Figure 4:**
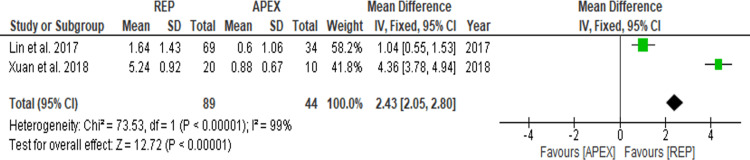
Root dentin wall thickness after APEX and REP (%). Lin et al. (2017) [19], Xuan et al. (2018) [21]. REP = regenerative endodontic procedure; APEX = apexification procedure

**Figure 5:**
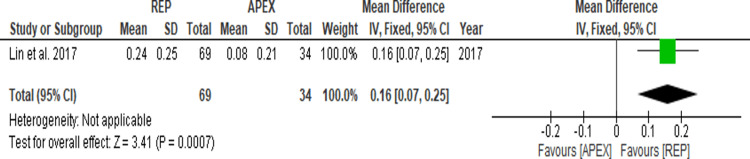
Root dentin wall thickness after APEX and REP (mm). Lin et al. (2017) [19]. REP = regenerative endodontic procedure; APEX = apexification procedure

Four of the included studies [17,18,20,22] presented the root dentin wall thickness results as a percentage (%), while the study by Lin et al. [19], uses millimeters (mm) to show the change. However, Xuan et al. [21] did not specify any measurements for the observed increase in root dentin wall thickness of the REP cases, and therefore the outcomes of this study could not be included in the meta-analysis. Figure 4 and Figure 5 illustrate the findings of the data analysis and forest plots that were created respectively. The MD in root dentin wall thickness (%) outcomes was 10.94 (95% CI = 7.01-14.88; p < 0.00001), while the MD in root dentin wall thickness (mm) outcome of the Lin et al. [18] study was 0.16 (95% CI = 0.07-0.25; p = 0.0007), which differed statistically.

Discussion

This systematic review aimed to evaluate the outcomes of revitalization and apexification procedures and the ability of the root length and dentin wall thickness development in immature teeth with pulp necrosis and/or apical periodontitis.

Revitalization is a process that seeks to generate new pulp-like tissue within the root canal to regenerate the tooth pulp and lower the likelihood of tooth loss [24] This may be one of the primary reasons contributing to the noteworthy outcomes seen with REP, particularly enhancing the root length and dentinal wall thickness compared to apexification. Apexification is considered a more intrusive technique, as the REP protocol mandates the placement of the materials in the coronal third of the root, rather than the periapical area [5]. However, the success of the treatment is heavily reliant on patient adherence. Alternatively, apexification is characterized as a technically challenging and expensive procedure, along with the achievement of an apical barrier being unpredictable [4].

All included studies in this review indicated that regardless of the different protocols followed, REP resulted in a significant increase in both root length and dentin wall thickness. Alternatively, apexification procedures demonstrated some impact on the development of root length, although the effect was not as significant as seen in REP cases [17-22]. However, there was no apparent improvement in root dentin wall thickness, as half of the analyzed studies showed no increase or even a reduction in root thickness [20-22].

Most of the available literature mentioning REPs involves the induction of the bleeding to allow the resulting BC to serve as a scaffold for revitalization. Caleza-Jiménez et al. [20] highlighted the growth factors contained in a BC can be attributed to the continuous root growth. Inducing bleeding into the canal system through over-instrumentation involves rotating a pre-curved K-file 2 mm past the apical foramen. The objective is to fill the entire canal with blood up to the cemento-enamel junction. Although inducing bleeding is a relatively simple procedure, this approach poses its own challenges. Some of the primary difficulties are the formation of a BC and its properties as it contains cells that ultimately die and release toxic intracellular enzymes that can potentially be harmful to the survival of the stem cells [17]. Instead of the formation of a blood clot, an alternative approach involves utilizing platelet-rich plasma, platelet-rich fibrin, or autologous fibrin matrix [9]. The use of dental pulp stem cells as a scaffold in revitalization procedures has also been suggested in endodontic literature [4] and it coincides with the protocol used by Xuan et al. [21]. These guidelines have been used in procedures that aim to regenerate the dentin/pulp complex. However, there is clinical evidence suggesting a correlation between the age of the patient and the success of such procedures, as it was observed that dental pulp stem cells in older patients had a lower potential for regeneration when compared to the stem cells of younger patients [4].

According to AAE clinical considerations on REP, TAP can be used as an intracanal medication. Clinicians should mix a balanced blend of ciprofloxacin, metronidazole, and minocycline at a 1:1:1 ratio, achieving a final concentration ranging from 1 to 5 mg/mL. Some literature suggested that TAP could be beneficial in REP cases as it could be utilized to create a favorable setting for vascular and regenerative cell growth by eliminating bacteria from the root canal of necrotic teeth with incompletely formed roots. However, there is a strong link between the use of TAP and crown discoloration, as it appears to be the most frequently seen side effect of its use [17]. To reduce the staining effect, it is advisable to contemplate the sealing of the pulp chamber using a dentin bonding agent [9].

The meta-analysis showed a statistically significant increase in the root length in all examined teeth that were subjected to the revitalization procedures [17-22]. The root dentin wall thickness increase was also considered to be statistically significant in most of the cases that received the REP treatment [17-20,22].

However, it is crucial to comprehend that the results obtained from each study present limitations. Certain limitations were found that might impact the results presented in this study. These limitations include the difference in anesthetic solution, irrigation concentrations, and TAP mixture, as well as a difference in the treatment protocols of REP and apexification, which may have an impact on root maturation quantitative results. It is important to observe that the etiology of pulp necrosis factors plays a significant role in the choice of disinfection protocol and overall treatment outcomes. Moreover, the presence of apical periodontitis makes healing mechanisms more complicated [25]. Different studied teeth groups and a wide range of follow-up periods within the research may also have an impact on the conclusions drawn from the analyzed studies. Regardless, it is important to note that the authors did not proclaim whether these factors should be considered catalytic to the results.

The duration of the follow-up visits could affect the incidence of clinically significant changes in root dimensions. Longer monitoring periods may increase the likelihood of notable outcomes. It is worth mentioning that this systematic review comprises studies with variable follow-up durations, yet all studies had at least six-month follow-up appointments, during which concrete results were presented. Initial results in terms of root development can be observed and evaluated even after three months post-treatment [26].

## Conclusions

This systematic review showed both procedures to be credible treatment options for necrotic immature teeth. Apexification had a positive impact, to some extent, on the development of root length. Based on the findings of the meta-analysis, revitalization yielded a significantly greater increase in both root length and root dentin wall thickness and appears to be superior to the apexification technique in promoting root development.
